# Prevalence and determinants of anaemia in women of reproductive age in Sudan: analysis of a cross-sectional household survey

**DOI:** 10.1186/s12889-020-09252-w

**Published:** 2020-07-17

**Authors:** Khalid Abdelmutalab Elmardi, Ishag Adam, Elfatih Mohammed Malik, Tarig Abdalla Abdelrahim, Mousab Siddig Elhag, Abdalla Ahmed Ibrahim, Mariam Adam Babiker, Asma Hashim Elhassan, Hmooda Toto Kafy, Azza Tageldin Elshafie, Lubna Mohammed Nawai, Mujahid Sheikhedin Abdin, Stef Kremers

**Affiliations:** 1 Health Information, Monitoring and Evaluation and Evidence Department, Federal Ministry of Health, Khartoum, Sudan; 2 Department of Obstetrics and Gynecology, Unaizah College of Medicine and Medical Sciences, Qassim University, Unaizah, Saudi Arabia; 3 Faculty of Medicine, University of Khartoum, Khartoum, Sudan; 4 Communicable and Non-Communicable Diseases Control Directorate, Federal Ministry of Health, Khartoum, Sudan; 5 Directorate of Pharmacy, Federal Ministry of Health, Khartoum, Sudan; 6 Directorate General of Primary Health Care, Federal Ministry of Health, Khartoum, Sudan; 7 Department of Health Promotion, NUTRIM School of Nutrition and Translational Research in Metabolism, Faculty of Health, Medicine and Life Sciences, Maastricht, The Netherlands

**Keywords:** Anaemia, Camps, Haemoglobin, Logistic regression, Malaria, Pregnant women, Sudan, Women in reproductive age

## Abstract

**Background:**

Anaemia is a global health problem and women in reproductive age (WRA) are amongst the most affected population. Its consequences include low birth weight and maternal mortality. This study aimed to assess the prevalence of anaemia and to identify its determinants in Sudanese women in reproductive age.

**Methods:**

A population-based cross-sectional study was conducted in Sudan in 2016. A multi-stage stratified cluster sampling design was executed with consideration of rural population, urban population, and internally displaced persons/refugees camps residents. All women in reproductive age (15–49 years), classified by pregnancy status, in the targeted households were surveyed and personal characteristic data were collected. Their haemoglobin level and malaria infection (using rapid diagnostic test, RDT) were assessed. The World Health Organization (WHO) haemoglobin level cut-off for defining anaemia and severe anaemia in pregnant and non-pregnant women was used. Logistic regression analyses were performed.

**Results:**

A total of 4271 women (WRA) of which 421 (9.9%) pregnant women (PW) were included in the study. The overall anaemia prevalence in WRA was 35.6%. It was 36.0 and 35.5% in PW and non-pregnant women (NPW), respectively. The average haemoglobin level was found to be 113.9 g/L (SD 16.3) and 123.2 g/L (SD 15.7) for PW and NPW respectively. Severe anaemia prevalence was 1.2% in each group. In the logistic regression model, anaemia was associated with malaria infection in PW (aOR 4.100, 95%CI 1.523–11.039, *p* = 0.003), NPW (aOR 2.776, 95%CI 1.889–4.080, *p* < 0.001), and WRA (aOR 2.885, 95%CI 2.021–4.119, *p* < 0.001). Other identified determinants of anaemia in NPW was living in camps (aOR 1.499, 95%CI 1.115–2.017, *p* = 0.007) and in WRA was being in the poorest economic class (aOR 1.436, 95%CI 1.065–1.936, *p* = 0.018).

**Conclusions:**

Anaemia is a public health problem in Sudan. The study supported the association between malaria infection and anaemia, but not with low and moderate malaria transmission areas. Resources need to be allocated for all anaemic populations with special attention for the populations in most need and interventions need to be implemented based on local variations. Malaria control interventions, specifically case management, may have a major impact in reducing anaemia prevalence in low to moderate malaria transmission areas.

## Background

Anaemia is a world-wide problem, but affecting more the low and middle-income countries with young children and pregnant women as the most affected population groups [1, 2]. The latest World Health Organization (WHO) report on anaemia estimated that 32.4 million pregnant women (PW) and 496.3 million non-pregnant women (NPW) worldwide were anaemic in 2011, giving a total of 528.7 million women of reproductive age (WRA). Out of those, 19.4 million NPW and 0.8 million PW were estimated as having severe anaemia, which sums up to a total of 20.2 million WRA [1, 2].

Causes of anaemia include blood loss, infections, acute and chronic diseases, micronutrient deficiencies, splenomegaly, and haemoglobinopathies [3, 4]. Among these, iron deficiency contributes to around 50% of the anaemia problem [2, 5]. On top of its social and economic development impact, the effect of anaemia as a contributing factor for maternal and perinatal adverse effect is reported. Evidence has documented the association between anaemia and postpartum haemorrhage, preterm labour, low birth weight, small for gestational age babies, and perinatal death [6–8]. Iron deficiency anaemia was found to affect work productivity and hence could lead to a loss of 1.3% of the gross domestic product [9].

The magnitude of the anaemia problem is classified based on its prevalence into mild public health problem when the level of anaemia prevalence is less than 20%, moderate public health problem if the prevalence is between 20% and less than 40%, and severe public health problem if it is equal to or more than 40% [10]. The global community set a target of 50% reduction of anaemia in WRA by 2025 as part of the comprehensive implementation plan on maternal, infant and young child nutrition [11]. Progress towards this target needs reliable statistics and monitoring systems at the country level. In Sudan, most of the recent data regarding anaemia are based on small-scale localized studies or estimations and many of these are health facility-based. Findings from these localized studies showed huge variation in anaemia prevalence (26.2 to 80.3%) [12–15]. Limitations of these studies stand a barrier against the country to use these figures as a base to monitor the progress. Micronutrient supplementation, promotion of dietary diversification and fortification, optimal hygiene, and infection control are among anti-anaemia interventions adopted in Sudan [16]. This study was done in Sudan to establish a national baseline figure for anaemia in WRA as well as to identify its determinants to better design interventions towards the neediest communities.

## Methods

This cross-sectional study was conducted in Sudan in 2016. Sudan is a low-middle income African country with 40.852 million US$ GDP per capita as estimated for the year 2018 [17]. The country with space of 1,882,000 Sq. Kilometres is administratively divided into 18 states. Some areas in Darfur region, South Kordofan and Blue Nile states are affected by civil conflict and insecurity which may affect accessibility. The country has 8 camps for internally displaced persons (IDPs) and is hosting 22 refugees’ camps which are located in 5 Darfurs, South Kordofan, West Kordofan, White Nile, Gedaref, and Kassala states. Refugees are mostly from countries such as Eritrea, Ethiopia and South Sudan. The total population for 2018 is 39.28 million, the life expectancy at birth is 59.8 years, and the annual growth rate is 2.8% [18]. The urban population represents 32.7% of the population, WRA are 25.7%, and the average household members are five [19].

A population-based household survey to measure the prevalence of anaemia in WRA (15–49 years), disaggregated by pregnancy status, was combined with a malaria indicator survey and carried out on November 2016 just after the rainy season. A multi-stage stratified cluster sampling technique was used to select the study clusters using the number of households and population in popular administrative units (PAUs) based on the 2008 census projected to 2016. All (8) internally displaced persons (IDP) camps and 4 randomly selected refugees camps were chosen for the study. Out of the national number of clusters calculated (509 clusters), the number of camps clusters has been assigned following the size of the population resident in these camps. Then, remaining clusters have been allocated equally to states. Within states, the allocation was in proportionate to rural/urban population ratios. In each cluster of more than 150 households, segmentation was done. Then, in a one randomly selected segment, 20 households were randomly selected. In each household, all WRA were enrolled in the survey. All households and participants were assured of their right to participate in this study and written consent was obtained from them before enrolment. Ethical clearance for this study was obtained from the Sudan federal ministry of health, ethical and technical review board.

Data from households were collected in Arabic language using a questionnaire designed in a digital personal data assistant (PDA) device by trained health personnel. Locally developed web-based survey application with its data validation functions was used for data collection. A finger-prick capillary blood sample was taken by trained laboratory assistants from study participants for testing of haemoglobin level using field battery-operated device (HemoCue® 301+ analyser from Radiometer Group) and for malaria testing using *Plasmodium falciparum* and *Plasmodium vivax* specific histidine-rich protein II malaria rapid diagnostic test (RDT) (SD BIOLINE Malaria Ag Pf/Pv® from STANDARD DIAGNOSTICS INC/ SD).

In this study, the WHO standard cut-off points for anaemia and severe anaemia for WRA is used [20–22]. Any PW with a haemoglobin level of less than 110 g/L is considered anaemic and with a haemoglobin level of less than 70 g/L is considered severely anaemic. On the other hand, any NPW with a haemoglobin level of less than 120 g/L was classified as having anaemia and < 80 g/L was classified as having severe anaemia. Anaemia in PW and NPW as per each group definition is combined to give the overall anaemia in WRA. The same was applied for severe anaemia.

Malaria infection is considered in the study when malaria parasite is identified in the study population using malaria rapid diagnostic test. Study participants who were found to be positive for malaria were treated according to the standard treatment guidelines while anaemic individuals were referred to the nearest health facility for medical attention. Using malaria parasite prevalence in children aged 2 to less than 10 years old, states that showed a parasite prevalence of less than 10% are considered as malaria hypo-endemic and those with a prevalence of 10% to less than 50% are considered malaria meso-endemic ones [23, 24]. No state demonstrated a malaria parasite prevalence of 50% or more to be classified as hyper-endemic. Wealth index was generated to provide a proxy comparable indicator of the household wealth status. The wealth index was calculated using principal component analysis (PCA) based on ownership of durable goods, household living condition, and level of household head education [25]. Variables included in PCA were restructured and recoded into multiple binary variables (0 = no/not present, and 1 = yes/ present). Based on the predicted wealth index, households were ranked and disaggregated into 5 equal quintiles, with the lowest wealth quintile stands for the poorest class of the population and the highest stands for the richest.

### Statistical analysis

The dependent variable in this study is the anaemia as per the definition described above. Independent variables included pregnancy status, age, type of residency, education level, wealth status, sanitation facility, water source, listening to radio (as a channel for health education), having health insurance, having malaria infection, and the level of malaria endemicity.

The analysis was done using SPSS 21. Descriptive analyses were done to describe the data and to demonstrate prevalence among PW, NPW and WRA. Mean haemoglobin level and standard deviation (SD) were generated and compared between PW and NPW and within anaemic sub-groups using T-test and ANOVA. Chi squire test was used to assess statistical differences in categorical data and T-test for continuous data. Then, multivariable analysis was done to examine the association between independent variables and anaemia (the outcome) as well as the strength of the association. Only independent variables that demonstrate a statistically significant effect on anaemia and/or on mean haemoglobin level in the bivariate analysis were further taken for the multivariable analysis. The logistic regression model was built based on a backward (likelihood Ratio) stepwise method. Original haemoglobin level variable was used to check for the absence of multicollinearity among independent variables. A variable that showed no statistical significance at *p* < 0.05 was not selected to be entered in the model. Odds Ratios with its 95% confidence intervals (95%CI) and P. values were reported. P. value of less than 0.05 was considered significant.

## Results

In this survey, a total of 4271 WRA were examined for their haemoglobin level. Of them, 1385 (32.4%) were in urban areas, 2654 (62.1%) were in rural areas and 232 (5.4%) were in camps. The PW involved in the study were 105 in urban areas, 295 in rural areas and 24 in camps giving a total of 421 (9.9%) PW. The mean (SD) of the age among PW was 27.1 (6.6) and it was 28.3 (9.0) years in NPW (*p* < 0.001).

### Anaemia prevalence

The overall prevalence of anaemia in WRA was 35.6%, with a prevalence of 36.1 and 35.5% in PW and NPW, respectively. The mean (SD) haemoglobin level was 113.9 (16.3) g/L and 123.2 (15.7) g/L in PW and NPW, respectively. Table 1 below shows the prevalence of anaemia, average haemoglobin levels, and prevalence of severe anaemia, by pregnancy status, between states and among camps, rural and urban residents. Anaemia in pregnancy is a severe public health problem in Khartoum, Kassala, South Kordofan, White Nile, West Kordofan, and South Darfur states and it is only a mild health problem in North Darfur State. On the other hand, anaemia in NPW was a severe health problem in Central Darfur, Kassala, Northern, and Khartoum states.

**Table 1:** Anaemia prevalence and mean haemoglobin level in women of reproductive age by pregnancy status, place of residence, and type of place of residence, Sudan, 2016

Anaemia prevalence and mean haemoglobin level										
**Variables**		**Pregnant women**			**Non-pregnant women**			**Women in reproductive age (pregnant and non-pregnant women combined)**		
		**Anaemia prevalence**	**Severe anaemia prevalence**	**Total number**	**Anaemia prevalence**	**Severe anaemia prevalence**	**Total number**	**Anaemia prevalence**	**Severe anaemia prevalence**	**Total number**
				**Average Haemoglobin level (g/L (SD))**			**Average Haemoglobin level (g/L (SD))**			
**SUDAN**	n	**152**	**5**	**421**	**1367**	**45**	**3850**	**1519**	**50**	**4271**
	%	**36.1**	**1.2**	**113.9 (16.3)**	**35.5**	**1.2**	**123.2 (15.7)**	**35.6**	**1.2**	**–**
**Place of residence (State)**										
**Northern**	n	5	0	17	97	1	220	102	1	237
	%	29.4	0.0	113.7 (12.2)	44.1	0.5	119.3 (14.2)	43.0	0.4	–
**River Nile**	n	5	0	22	79	0	286	84	0	308
	%	22.7	0.0	119.5 (12.2)	27.6	0.0	125.8 (13.3)	27.3	0.0	–
**Red Sea**	n	4	0	18	71	1	187	75	1	205
	%	22.2	0.0	112.4 (8.3)	38.0	0.5	121.4 (13.8)	36.6	0.5	–
**Kassala**	n	24	1	44	136	8	279	160	9	323
	%	54.5	2.3	110.1 (17.1)	48.7	2.9	117.7 (17.0)	49.5	2.8	–
**Gedarif**	n	6	0	28	67	5	291	73	5	319
	%	21.4	0.0	120.8 (25.6)	23.0	1.7	127.7 (15.3)	22.9	1.6	–
**Khartoum**	n	18	0	31	127	5	304	145	5	335
	%	58.1	0.0	108.9 (16.0)	41.8	1.6	120.4 (16.2)	43.3	1.5	–
**Gezira**	n	7	0	26	103	2	295	110	2	321
	%	26.9	0.0	117.5 (12.5)	34.9	0.7	123.6 (13.0)	34.3	6	–
**White Nile**	n	9	0	20	82	5	232	91	5	252
	%	45.0	0.0	113.1 (13.7)	35.3	2.2	123.0 (15.9)	36.1	2.0	–
**Sinnar**	n	6	0	17	96	2	255	102	2	272
	%	35.3	0.0	110.0 (13.3)	37.6	0.8	122.3 (14.8)	37.5	0.7	–
**Blue Nile**	n	17	0	45	56	0	207	73	0	252
	%	37.8	0.0	114.2 (18.3)	27.1	0.0	124.3 (12.8)	29.0	0.0	–
**North Kordofan**	n	6	0	19	74	1	230	80	1	249
	%	31.6	0.0	113.3 (16.8)	32.2	0.4	124.0 (14.5)	32.1	0.4	–
**South Kordofan**	n	8	0	15	38	0	142	46	0	157
	%	53.3	0.0	113.1 (18.1)	26.8	0.0	127.7 (14.4)	29.3	0.0	–
**West Kordofan**	n	4	0	9	30	1	95	34	1	104
	%	44.4	0.0	112.1 (14.6)	31.6	1.1	125.7 (18.5)	32.7	1.0	–
**North Darfur**	n	5	1	27	74	0	213	79	1	240
	%	18.5	3.7	117.7 (17.3)	34.7	0.0	125.6 (15.7)	32.9	0.4	–
**West Darfur**	n	7	0	23	39	2	149	46	2	172
	%	30.4	0.0	118.0 (15.6)	26.2	1.3	127.1 (15.7)	26.7	1.2	–
**South Darfur**	n	10	2	24	87	4	224	97	6	248
	%	41.7	8.3	109.8 (23.2)	38.8	1.8	124.5 (18.4)	39.1	2.4	–
**Central Darfur**	n	8	1	21	84	5	163	92	6	184
	%	38.1	4.8	111.8 (21.2)	51.5	3.1	118.0 (16.8)	50.0	3.3	–
**East Darfur**	n	3	0	15	27	3	78	30	3	93
	%	20.0	0.0	122.2 (15.0)	34.6	3.8	124.1 (23.8)	32.3	3.2	–
*p. value*		0.033*	*0.371*	*0.199*	< 0.001*	0.007*	< 0.001*	< 0.001*	0.003*	–
**Type of place of residence (area classification)**										
**IDPs/Refugees Camps**	n	10	2	24	95	6	208	105	8	232
	%	41.7	8.3	109.2 (23.1)	45.7	2.9	121.0 (18.3)	45.3	3.4	–
**Urban**	n	31	1	105	461	18	1280	492	19	1385
	%	29.5	1.0	118.0 (19.8)	36.0	1.4	123.2 (16.3)	35.5	1.4	–
**Rural**	n	111	2	292	811	21	2362	922	23	2654
	%	38.0	0.7	113.2 (15.5)	34.3	0.9	123.5 (15.2)	34.7	0.9	–
*p. value*		*0.252*	0.004*	0.018*	0.004*	0.023*	*0.082*	0.006*	0.001*	–

*p*. value was calculated based on *x*^*2*^ test for categorical data (anaemia prevalence) and with t-test for continuous data (average haemoglobin level)

*p*. values with statistical significance (*p* < 0.05) is denoted with *

Based on this study anaemia is classified as severe public health problem in both PW and NPW residents in camps (PW: 41.7%, NPW: 45.7%) compared to rural (PW: 38.0%, NPW: 34.3%) and urban (PW: 29.5%, NPW: 36.0%) residents (Table 1). This variation in prevalence is only statistically significant in NPW (*p* = 0.004) but not PW (*p* = 0.252).

### Determinants of anaemia in pregnant women

There were no significant statistical differences in the type of place of residence, level of education, economic status, sanitation facility used, source of drinking water, listening to the radio, having health insurance, or variations in the level of malaria endemicity between anaemic PW and non-anaemic PW according to findings of the bivariate analysis. There was a higher prevalence of anaemia (69.6%) with malaria-infected PW compared to non-infected PW (*p* = 0.001). The mean of the haemoglobin level in PW was significantly lower in Camps residents (109.2 g/L (SD 23.1); *p* = 0.018), and in women using unsafe sources of drinking water (113.3 g/L (SD16.7); *p* = 0.044 for open source), and in women with malaria infection (100.8 g/L (SD18.9) *p* < 0.001) (Tables 1 and 2). The final model of the regression analysis has identified that only malaria infection is associated with anaemia in pregnancy (adjusted odds ratio (aOR) 4.100; 95%CI: 1.523–11.039; *p* = 0.005).

**Table 2:** Factors affecting anaemia prevalence and mean haemoglobin level in women of reproductive age, Sudan, 2016

**Variables**		**Pregnant women**		**Non-pregnant women**		**Women in reproductive age (pregnant and non-pregnant women combined)**	
		**Anaemia prevalence**	**Total number**	**Anaemia prevalence**	**Total number**	**Anaemia prevalence**	**Total number**
			**Average Haemoglobin level (g/L (SD))**		**Average Haemoglobin level (g/L (SD))**		
**Level of education**							
**No formal education**	n	60	157	400	1047	460	1204
	%	38.2	114.6 (18.3)	38.2	122.4 (16.0)	38.2	–
**Primary/religious education**	n	67	176	537	1587	604	1763
	%	38.1	112.8 (18.0)	33.8	123.7 (15.1)	34.3	–
**Secondary education**	n	15	53	259	764	274	817
	%	28.3	116.9 (12.0)	33.9	123.8 (16.6)	33.5	–
**Above secondary education**	n	10	35	171	452	181	487
	%	28.6	115.8 (14.7)	37.8	122.6 (16.0)	37.2	–
*p. value*		*0.414*	*0.407*	*0.066*	*0.118*	*0.072*	–
**Wealth index**							
**Wealthiest**	n	21	76	384	1085	405	1161
	%	27.60%	117.8 (17.3)	35.40%	122.9 (15.1)	35.50%	–
**High**	n	33	99	336	1008	369	1107
	%	33.30%	115.7 (19.0)	33.30%	123.8 (15.5)	33.30%	–
**Middle**	n	29	81	221	652	250	733
	%	35.80%	113.6 (16.1)	33.90%	123.9 (16.7)	34.10%	–
**Low**	n	33	83	186	535	219	618
	%	39.80%	112.7 (15.6)	34.80%	122.8 (15.8)	35.40%	–
**Poorest**	n	36	82	240	570	276	652
	%	43.90%	111.2 (20.4)	42.10%	122.5 (16.1)	42.30%	–
*p. value*		*0.254*	*0.117*	0.008*	*0.324*	0.003*	*–*
**Sanitation type**							
**Safe sanitation**	n	24	59	298	800	322	859
	%	40.7	114.3 (14.7)	37.3	122.4 (15.8)	37.5	–
**Unsafe sanitation**	n	82	226	718	2128	800	2354
	%	36.3	113.9 (17.8)	33.7	123.6 (15.7)	34	–
**Open defecation**	n	44	125	339	864	383	989
	%	35.2	114.5 (17.9)	39.2	122.8 (15.7)	38.7	–
*p. value*		*0.764*	*0.954*	0.011*	*0.145*	0.017*	*–*
**Source of drinking water**							
**Piped to the house or bottled water**	n	13	55	286	786	299	*841*
	%	23.6	119.6 (14.6)	36.4	122.7 (15.4)	35.6	*–*
**Piped to public area**	n	50	131	452	1309	502	*1440*
	%	38.2	113.4 (18.9)	34.5	123.3 (15.5)	34.9	*–*
**Open source**	n	81	212	588	1634	669	*1846*
	%	38.2	113.3 (16.7)	36	123.5 (16.0)	36.2	*–*
*p. value*		*0.114*	0.044*	*0.616*	*0.487*	*0.714*	*–*
**Frequently listen to the radio**							
**Yes**	n	49	117	378	1105	427	1222
	%	41.9	114.3 (20.0)	34.2	123.7 (15.4)	34.9	–
**No**	n	103	301	986	2734	1089	3035
	%	34.2	114.1 (16.1)	36.1	123.1 (15.9)	35.9	–
*p. value*		*0.147*	*0.884*	*0.28*	*0.282*	*0.572*	*–*
**Have health insurance**							
**Yes**	n	39	129	484	1333	523	1462
	%	30.2	116.2 (14.4)	36.3	123.0 (16.9)	35.8	–
**No**	n	113	292	883	2517	996	2809
	%	38.7	113.3 (18.3)	35.1	123.4 (15.8)	35.5	–
*p. value*		*0.1*	*0.114*	*0.457*	*0.516*	*0.84*	*–*
**Malaria infection (parasitaemia)**							
**Positive**	n	16	23	72	118	88	141
	%	69.6	100.8 (18.9)	61.0	115.7 (13.6)	62.4	–
**Negative**	n	136	397	1292	3722	1428	4119
	%	34.3	114.9 (16.8)	34.7	123.5 (15.7)	34.7	–
*p. value*		0.001*	< 0.001*	< 0.001*	< 0.001*	< 0.001*	–
**Level of malaria endemicity**							
**Meso-endemic (PR2–10 = 10- < 50%)**	n	33	81	178	512	211	593
	%	40.7	113.4 (18.8)	34.8	123.2 (15.1)	35.6	–
**Hypo-endemic (PR2–10 = < 10%)**	n	119	340	1189	3338	1308	3678
	%	35.0	114.4 (16.9)	35.6	123.2 (15.8)	35.6	–
*p. value*		*0.334*	*0.633*	*0.707*	*0.977*	*0.993*	*–*

*p*. value was calculated based on *x*^*2*^ test for categorical data (anaemia prevalence) and with t-test for continuous data (average haemoglobin level)

*p*. values with statistical significance (*p* < 0.05) is denoted with *

### Determinants of anaemia in non-pregnant women

There were no significant statistical differences between anaemic and non-anaemic NPW’s age, level of education, source of drinking water, listening to the radio, having health insurance and level of malaria endemicity as revealed by the bivariate analysis. However, anaemia in NPW was found to be associated with the type of place of residence (higher prevalence in camps residents (45.7%); *p* = 0.004), wealth index (higher prevalence among the poorest (42.1%); *p* = 0.008), type of sanitation facility (higher prevalence among those practising open defecation (39.2%); *p* = 0.011), and malaria infection (higher prevalence among NPW with a positive malaria test 61.0%; *p* < 0.001). Compared to this, haemoglobin level in NPW was found to be associated with the type of place of residence (lower levels in camps residents (121.0 g/L (SD18.3)); *p* = 0.023), and with malaria infection (lower level in NPW with positive malaria test (115.7 g/L (SD13.6)); *p* < 0.001) (Tables 1 and 2). The multivariable logistic regression final model revealed an association between anaemia in NPW and type of place of residence (*p* = 0.016), type of sanitation facility (*p* = 0.005), and malaria infection (p < 0.001) (Table 3). Anaemia odds were higher in camps NPW by almost 50% compared rural residents (aOR 1.499; 95%CI 1.115–2.017; P 0.007) while there was no statistically significant difference in odds of urban residents compared to rural residents (aOR 1.134; 95%CI 0.965–1.333; P 0.126). Despite that the model demonstrated an overall association between anaemia in NPW and type of sanitation facility used, there was no statistical significant difference in the odds of those using unsafe sanitation facility (aOR 0.854; 95%CI 0.714–1.022; P 0.085) or those with no sanitation facility (open defecation) (aOR 1.122; 95%CI 0.897–1.404; P 0.313) when compared to those who use safe sanitation facilities. The odds of anaemia in NPW infected with malaria was more than 1.7 times higher than in non-infected women (aOR 2.776; 95%CI 1.889–4.080; *P* < 0.001).

**Table 3:** Regression analysis for factors associated with anaemia in non-pregnant women, Sudan, 2016

**Variables**	**Frequency**	**Unadjusted analysis**		**Adjusted analysis**	
		**cOR**^**a**^**(95%CI)**	***P*. value**^**‡**^	**aOR**^**b**^**(95%CI)**	***p*. value**^**‡**^
**Type of place of residence (area classification)**					
**Rural**	2291	1	**0.015**	1	**0.016**
**Urban**	1231	1.135 (0.958–1.346)	0.144	1.134 (0.965–1.333)	0.126
**IDP/Refugee Camps**	200	1.505 (1.110–2.040)	0.008	1.499 (1.115–2.017)	0.007
**Wealth index**					
**Wealthiest**	1052	1	**0.193**		
**High**	972	0.972 (0.802–1.178)	0.772		
**Middle**	627	0.967 (0.763–1.225)	0.778	–	–
**Low**	521	0.936 (0.714–1.227)	0.632		
**Poorest**	550	1.289 (0.941–1.767)	0.114		
**Sanitation type**					
**Safe sanitation facility**	773	1	**0.174**	1	**0.005**
**Unsafe sanitation facility**	2096	0.860 (0.713–1.038)	0.116	0.854 (0.714–1.022)	0.085
**Open defecation**	853	0.983 (0.735–1.313)	0.906	1.122 (0.897–1.404)	0.313
**Malaria infection (parasitaemia)**					
**Negative**	3608	1	**< 0.001**	1	**< 0.001**
**Positive**	114	2.756 (1.871–4.058)	< 0.001	2.776 (1.889–4.080)	< 0.001

^a^ cOR: Crude Odd Ratio. ^b^ aOR: Adjusted Odd Ratio

‡ *p*. values in bold reflect the overall exposure and are derived from the likelihood ratio test. p. value for variables with multiple exposure level are reported from the Wald test

Goodness-of-fit test (Hosmer and Lemeshow) for the adjusted final model for non-pregnant women *X*^*2*^ 0.210 (4 degrees of freedom; *p*. value = 0.995)

### Determinants of anaemia in women in reproductive age

When anaemia status was combined in PW and NPW, the univariate analysis showed no significant statistical differences between anaemia in WRA and age, level of education, source of drinking water, listening to the radio, having health insurance, and level of malaria endemicity. A higher prevalence of anaemia was observed in WRA in camps residents (45.3%; *p* = 0.006), among the poorest (42.3%; *p* = 0.003), among those who were practising open defecation (38.7%; *p* = 0.017), and among those with malaria infection (62.4%; *p* < 0.001) (Tables 1 and 2). The logistic regression final model showed that anaemia in WRA was associated with the type of place of residence (*p* = 0.033), wealth index (*p* = 0.010), and malaria infection (p = 0.003). Pregnancy status was not found to have an association with anaemia in WRA (*p* = 0.916) (Table 4). Odds of anaemia in WRA resident in camps were higher (aOR 1.397; 95%CI 1.048–1.863; P 0.023) compared to Rural WRA, but the association was not significant for urban residents (aOR 1.095; 95%CI 0.931–1.289; P 0.274). For the economic class of WRA expressed as the wealth index, the odds of anaemia in the poorest women was increased (aOR 1.436; 95%CI 1.065–1.936; P 0.018) when compared to the wealthiest group. The odds of anaemia in WRA that have malaria infection was higher by 1.9 times (OR 2.885; 95%CI 2.021–4.119; *p* < 0.001) compared to those with non-malaria infection.

**Table 4:** Regression analysis for factors associated with anaemia in women in reproductive age, Sudan, 2016

Women in reproductive age (pregnant and non-pregnant women combined)					
**Variables**	**Frequency**	**Unadjusted analysis**		**Adjusted analysis**	
		**cOR**^**a**^**(95%CI)**	***p*. value**^**‡**^	**aOR**^**b**^**(95%CI)**	***p*. value**^**‡**^
**Type of place of residence (area classification)**					
**Rural**	2571	1	**0.052**	1	**0.033**
**Urban**	1334	1.095 (0.931–1.289)	0.274	1.095 (0.931–1.289)	0.274
**IDP/Refugee Camps**	244	1.398 (1.048–1.863)	0.022	1.397 (1.048–1.863)	0.023
**Pregnancy status**					
**Pregnant**	407	1	**0.916**	–	–
**Non-pregnant**	3722	0.988 (0.796–1.227)	0.916		
**Wealth index**					
**Wealthiest**	1126	1	**0.081**	1	**0.010**
**High**	1070	0.995 (0.827–1.198)	0.961	0.996 (0.828–1.198)	0.963
**Middle**	702	1.026 (0.818–1.286)	0.825	1.026 (0.819–1.286)	0.823
**Low**	601	1.041 (0.806–1.344)	0.760	1.041 (0.806–1.345)	0.765
**Poorest**	630	1.435 (1.064–1.935)	0.018	1.436 (1.065–1.936)	0.018
**Sanitation type**					
**Safe sanitation facility**	831	1	**0.121**		
**Unsafe sanitation facility**	2320	0.827 (0.691–0.991)	0.040	–	–
**Open defecation**	978	0.850 (0.645–1.120)	0.249		
**Malaria infection (parasitaemia)**					
**Negative**	3992	1	**0.007**	1	**0.003**
**Positive**	137	2.883 (2.019–4.117)	< 0.001	2.885 (2.021–4.119)	< 0.001

^a^ cOR: Crude Odd Ratio. ^b^ aOR: Adjusted Odd Ratio

‡ *p*. values in bold reflect the overall exposure and are derived from the likelihood ratio test. p. value for variables with multiple exposure level are reported from the Wald test

Goodness-of-fit test (Hosmer and Lemeshow) for the adjusted final model for women of reproductive age *X*^*2*^ 4.633 (7 degrees of freedom; p*. value* = 0.705)

## Discussion

Anaemia in WRA is a worldwide health problem. This study aimed to measure the prevalence of anaemia in WRA in Sudan and to identify its determinants. Findings of this study demonstrated a high level of anaemia prevalence in the country and sub-country. Estimation for Sudan, as cited by the WHO for the year 2011, showed anaemia prevalence of 25 and 31% with a mean haemoglobin level of 116 g/L and 126 g/L for PW and NPW respectively [1, 2]. Our results show slightly higher levels of anaemia compared to previous WHO 2011 estimates for Sudan. Result of our study may play a role in validating country figures in the future. Both the WHO country estimates and our study values classified the problem of anaemia in Sudan to a moderate public health problem. On the other hand, this study showed a lower prevalence of anaemia among PW compared to an overall estimate of 53% generated by a systematic review of published research from Sudan [26]. This variation may be because most of the studies included in the systematic review were health facility-based compared to this population-based study. Based on the results of this study, the trend of anaemia in Sudan in WRA is markedly lower (35.6%) compared to six states average population survey of 47.8% performed in 1995 [27]. It is hard to conclude the trend of anaemia in Sudan over time based on these three examples discussed above. Differences in anaemia prevalence may be due to variations in the method of estimation, targeted population, and sample size. However, we feel that our study provides valid insight into population values among women in reproductive age in Sudan, as a result of our sampling and measurement methodologies. The situation in Sudan is not different from many countries in the region or outside of the region. For example, the prevalence of anaemia in PW is 39.8% in China [5]. In Ethiopia, the prevalence of anaemia in WRA was estimated at 23% [28]. Another good performing African country, Rwanda showed a prevalence of anaemia in WRA of 19.2% [2, 29]. Despite this low estimate in Rwanda and the noticeable improvement in maternal health services, the actual situation was showing an increasing trend in anaemia prevalence from a previous level of 17% in 2010 with huge variations at subnational level [29]. Both Ethiopia and Rwanda figures are away from a prevalence of 35.6% in this study. Despite the low number of cases identified as severe anaemia in this study, the overall prevalence does not look different from the WHO estimation for the country and the regional office figure [1, 2].

Subnational variation in anaemia prevalence is documented in many countries [29–32]. This rings the bell for the need to directing available resources to communities in high need as well as to design interventions at local levels based on the gaps and needs. A quite high number of states, in this study, were classified in the category of severe public health problem for anaemia in PW and NPW which should receive high attention and rapid actions. It is noticed in this study that anaemia prevalence in PW is markedly higher than that in NPW in many states and the situation is opposite in many others. Further work may be needed to explore why this is the case in order to inform future interventions.

NPW and WRA in camps tended to be more prone to have anaemia compared to rural women as per the findings of this study. The special circumstances facing population living in camps (both IDPs and refugees) affecting their whole life including their health status. This study classified PW and NPW living in camps as affected by a severe public health problem. It is also noticed that NPW in rural areas look like as being less affected by anaemia compared to urban and camp women. Severe anaemia is seen with high prevalence both among PW and NPW in camps compared to the rural and urban population. Overall results of this study stand at giving more priority for PW and NPW at camps in regards to anti anaemia interventions. Many studies documented the high burden of anaemia in IDPs and Refugees camps [33–37].

Our findings of the association between malaria and anaemia were reported by several previous studies [29, 38]. Malaria has complicated pathophysiology in causing anaemia. Anaemia in malaria is mainly due to the removal of unparasitized red blood cells by the spleen as well as the destruction of parasitised erythrocytes as part of the schizogony process. These two processes are usually accompanied by erythropoiesis dysfunction [39–41]. With the fact that anaemia develops rapidly in malaria, the second process may have little effect [39, 40]. Immunological factors and mechanism play an important role in the development of anaemia in malaria which include innate, cell-mediated and humoral immune systems as well as a non-specific immune response [39, 40]. The development of severe anaemia is due to the failure of the bone marrow to recover from a previous insult as a result of repeated malaria infections [39, 41]. Both parasitised and unparasitised red blood cells become less deformable in severe malaria and thus removed by the spleen [41]. Despite that pregnant women in moderate and high malaria transmission areas are previously immune, they tend to experience asymptomatic *P. falciparum* malaria, and hence be more prone to the pathogenesis of anaemia. Placental parasitaemia may be present in the absence of peripheral parasitaemia and thus contribute to anaemia. In such settings, the effect of *P. falciparum* infection during pregnancy is affecting first pregnancies at the most [40, 42]. In Sudan, most of the states are classified as hypo-endemic malaria areas with few as meso-endemic [43]. Results of this study did not demonstrate the relationship between anaemia in PW and the level of malaria endemicity. So, the national authority has to revise the need for using IPTp as a strategy for malaria control in areas classified as meso-endemic in Sudan. On the other hand, timely access to malaria diagnosis and prompt case management may have a superior role in reducing anaemia on top of other malaria control interventions in low to moderate malaria transmission settings.

This study did not find an association between pregnancy status and anaemia. In a systematic review involved four countries from Africa anaemia, iron deficiency, and iron deficiency anaemia prevalence were found to be lower in PW compared to WRA [44]. The same situation was seen in Europe [45]. The case in the United States (US) does not prove this. In the US, iron deficiency was found to be higher in PW compared to NPW, but iron deficiency anaemia was found to be lower in PW compared to NPW [46].

The association between anaemia and parasitic infection is well established. Evidence showed good impact in reducing anaemia through interventions against helminths in high-risk communities [4]. This research studied the link between access to safe water and access to safe sanitation to anaemia. Safe water sources and safer sanitation facilities are considered as major interventions in reducing soil-transmitted helminths. Type of sanitation facility used was found in this study to be only associated with anaemia in NPW. However, there is no statistical difference in odds of anaemia among those using unsafe sanitation facilities or open defecation with those who have safe sanitation facilities. Access to safe water sources was found to be neither associated with anaemia in PW nor with anaemia in NPW.

In this study, the association between economic status and anaemia was found for WRA. Results show that the poorest are more affected by anaemia than the richest women. This research is supporting other findings [29]. A study in Myanmar, however, did not find an association between anaemia and wealth status [32].

Many studies showed the lack of association between anaemia in pregnancy and mothers’ age and level of education [12, 13, 29, 32]. Such a situation has been identified in this research among WRA including PW and NPW. In Rwanda, exposure to educational channels such as reading newspapers, listening to the radio, or watching television did not show any statistical difference in the prevalence of anaemia in pregnancy compared to unexposed [29]. Listening to the radio did however not appear to affect anaemia prevalence in PW and NPW in our study. Financial hardship on communities stands as a big barrier for accessibility to health services. Health insurance as a mean for overcoming this challenge served many beneficiaries. This study did not find any statistical difference of anaemia between those with health insurance and those without, both among PW and NPW. Some barriers may however still face the population with health insurance from getting the benefit of health insurance services. Such obstacles need to be elaborated and addressed to have financial protection available for all.

This study has various strengths and limitations. Study limitations include the low frequency of severe anaemia that limits further analysis to find out its determinants. Another issue is that the level of haemoglobin/anaemia neither adjusted for smoking nor the altitude. More is that pregnancy status in this study was not validated by any test or procedure and was depending on women response. This study did not address parity, previous obstetrical complications, or screening for haemoglobinopathies, enzymopathies, haematological disorders, or chronic diseases. Neither participants’ use of pica nor the use of iron or folic acid supplements were assessed in this study. The cross-sectional design of this study and the nature of the logistic regression model developed with a limited number of variables are the additional limitation of this study. One major strength of this study is that it is the first large-scale study that provided anaemia prevalence estimates for all states of the country. This will set real-life baseline estimates for monitoring progress in the fight against anaemia. Other study strengths include disaggregation of results by pregnancy status, coverage of IDPs/refugees camps population, the focus on determinants of anaemia, and presentation of results by both anaemia prevalence and haemoglobin level.

## Conclusions

With the level of anaemia prevalence identified in this study, WRA in Sudan are facing a challenging health problem, both at national and sub-national levels. The problem is severely affecting camps residents. The study established the association between malaria infection and anaemia. With the context of Sudan, low and moderate malaria transmission was not found to affect anaemia prevalence. Thus, early diagnosis and effective treatment of malaria cases are of utmost importance in such situation.

Concerning anaemia, Sudan health authority needs to consider subnational level variations and give priority to the people most in need when designing interventions and allocating resources. Achieving progress in access to prompt and effective malaria case management is likely to have a high impact on reducing the burden of anaemia. Improving living conditions as well as reducing poverty may contribute to improving the haemoglobin level of affected communities.

## Data Availability

The datasets used during the current study are available from the Communicable and Non-communicable diseases Control Directorates, Federal Ministry of Health, Sudan on reasonable request.

## Abbreviations

aOR: Adjusted Odd ratio
GDP: Gross domestic product
IDPs: Internally displaced persons
IPTp: Intermittent presumptive treatment for pregnant women
NPW: Non-pregnant women
OR: Odd ratio
PAUs: Popular administrative units
PCA: Principal component analysis
PDA: Personal data assistant
PW: Pregnant women
RDT: Rapid diagnostic test
SD: Standard deviation
US: The United States
WHO: World Health Organization
WRA: Women in reproductive age
95%CI: 95% confidence interval
